# Explainable AI for forensic speech authentication within cognitive and computational neuroscience

**DOI:** 10.3389/fnins.2025.1692122

**Published:** 2025-11-05

**Authors:** Zhe Cheng, Haitao Yang, Yingzhuo Xiong, Xuran Hu

**Affiliations:** ^1^Department of Criminal Investigation, Hunan Police Academy, Changsha, China; ^2^Criminal Investigation Police University of China, School of Public Security Information Technology and Intelligence, Shenyang, China; ^3^School of Electronic Engineering, Xidian University, Xian, China

**Keywords:** multimedia forensics, digital speech processing, authentic detection, explainable artificial intelligence, cognitive neuroscience

## Abstract

The proliferation of deepfake technologies presents serious challenges for forensic speech authentication. We propose a deep learning framework combining Convolutional Neural Networks (CNNs) and Long Short-Term Memory (LSTM) networks to improve detection of manipulated audio. Leveraging the spectral feature extraction of CNNs and the temporal modeling of LSTMs, the model demonstrates superior accuracy and generalization across the ASVspoof2019 LA and WaveFake datasets. Linear Frequency Cepstral Coefficients (LFCCs) were employed as acoustic features and outperformed MFCC and GFCC representations. To enhance transparency and trustworthiness, explainable artificial intelligence (XAI) techniques, including Grad-CAM and SHAP, were applied, revealing that the model focuses on high-frequency artifacts and temporal inconsistencies. These interpretable analyses validate both the models design and the forensic relevance of LFCC features. The proposed approach thus provides a robust, interpretable, and XAI-driven solution for forensic authentic detection.

## 1 Introduction

The development of digital speech technologies has greatly advanced daily applications, yet it also introduces new challenges, particularly in the context of forensic evidence collection. Digital audio data is increasingly vulnerable to sophisticated manipulation attacks, making it difficult to ensure the integrity of evidence (Reis and Ribeiro, 2024). In response, researchers have proposed passive audio evidence collection techniques, which analyze original audio to detect signs of tampering, assess the extent of manipulation, and ensure the fairness of digital evidence (Gibb et al., 2018). However, as digital speech manipulation technologies continue to evolve, they present new obstacles to these traditional techniques, necessitating ongoing innovation in forensic practices to maintain the credibility of digital evidence (Jiang et al., 2024).

Early attempts at audio manipulation were limited and often resulted in poor-quality audio that could be identified through traditional forensic analysis. These methods included editing audio segments using simple audio editors and altering audio hashes or device metadata to obscure the source (Nye et al., 1975; Furui, 2018; Gold et al., 2011). Although these early techniques were rudimentary, they helped establish initial standards for identifying manipulated audio, which have since been refined with advancements in forensic technology (Jiang et al., 2025a,b). As digital manipulation techniques become more sophisticated, it is essential to continuously improve forensic methods to counteract emerging threats, ensuring the reliability and fairness of digital evidence.

Recent advancements in deep learning have enabled the creation of highly realistic synthetic speech, posing significant challenges to systems like automatic speaker verification (ASV), which are widely used in finance, authentication, and security (Liu et al., 2019, 2025a). Malicious deepfakes, if used improperly, can easily deceive ASV or human auditory systems (HAS), leading to severe consequences such as fraud and financial losses (Poddar et al., 2017; Kemp, 1978; Voloshynovskiy et al., 2001). For example, in 2019, a deepfake of a German executive was used to defraud a UK subsidiary of a major company (Stupp, 2019). In 2024, a multinational firm lost millions due to deepfake fraud (Drew todd, 2024). As telecom fraud continues to rise, the application of deepfakes by criminals becomes more prevalent, highlighting the need for advanced detection methods to combat this growing threat (Aziz and Andriansyah, 2023).

In this paper, we focus on the use of spectral features in forensic digital audio analysis, applying time-frequency techniques to detect manipulated speech. We propose a CNN-LSTM neural network model to automatically screen audio clips from the ASVspoof2019-LA benchmark dataset. By analyzing the spectrogram representation of the audio, we jointly evaluate temporal and spectral properties to distinguish manipulated signals from natural ones. Preliminary results show the potential of our approach for forensic audio examination, with further optimization of network architectures and inclusion of auxiliary modal cues likely to enhance robustness. Our findings support the use of deep learning methods for passive evidence evaluation and call for further exploration in this area.

## 2 Related work

In traditional forensic voice comparison, passive evidence collection is often employed due to stringent legal procedural requirements (Broeders, 2001; Martinovic and Tripalo, 2017; Frumarová, 2022; Koenig and Lacey, 2015; Maher, 2009). After client engagement, forensic experts conduct spectral analysis, comparing questioned audio with known reference samples to identify similarities and differences. This process requires significant forensic expertise and time to yield scientifically valid conclusions. However, the rise of digital audio deepfakes, which can now be synthesized with low barriers to entry and disseminated widely, complicates forensic analysis. The adaptability of passive courtroom audio analysis necessitates the development of proactive defenses, such as automated identification algorithms, to address the growing threat of deepfake misuse. As fraudulent synthetic vocal media risks undermining institutional trust and causing public harm through deceptive misinformation, there is a clear need to advance evidence evaluation standards and technical solutions.

Deep learning-based fake audio detection algorithms focus on the core binary classification problem of distinguishing between natural and manipulated speech (Liu et al., 2025b, 2024). These algorithms typically use downstream classifiers that process acoustic features extracted by front-end models. Traditional systems rely on machine learning methods applied directly to these representations. Common techniques include Gaussian Mixture Models (GMMs) (Masood et al., 2022), Support Vector Machines (SVMs) (Kawa et al., 2022), and Probabilistic Linear Discriminant Analysis (PLDA) (Agarwal et al., 2021). GMMs, which combine multiple Gaussian classifiers through linear weighting, remain popular due to their fast training speed, high accuracy, and broad applicability. They ranked first in the ASVspoof 2015 Challenge for countermeasures against presentation attacks. SVMs are also widely used for their strong generalization and fast training capabilities, ranking second in ASVspoof 2015 (Rahman et al., 2022; Wu et al., 2014).

Recently, neural networks have become increasingly popular for fake audio detection, as they can learn higher-level features from acoustic representations. For example, Lavrentyeva et al. (2017) improved a Lightweight Convolutional Neural Network (LCNN) with softmax edge activations for logical access attack detection in ASVspoof 2019. Ravanelli and Yoshua (2018) combined Deep Convolutional and SincNet architectures to perform well on LA attacks, though their generalization to unknown methods was weaker. Yang et al. (2024) proposed a dual-branch structure for detecting forgery information. Despite these advancements, challenges remain in achieving robust fake audio detection across diverse and evolving manipulation techniques.

## 3 Method

### 3.1 Speech spectral analysis

Based on principles of synthetic audio generation, deepfake methods relying on text-to-speech and voice conversion require character-to-phoneme or phoneme-to-phoneme conversion prior to waveform reconstruction via concatenation of synthesized phonemes. However, concatenation inevitably introduces perturbations, distortions and misalignments, hindering deepfakes from accurately modeling natural audio temporal features. In the frequency domain, where deepfakes are commonly modeled with inverted filter coefficients (Liu et al., 2023, 2025c), interrupted fundamental frequencies, lack of smoothness, missing high frequency content and background noise are easily exhibited.

**Figure 1:**
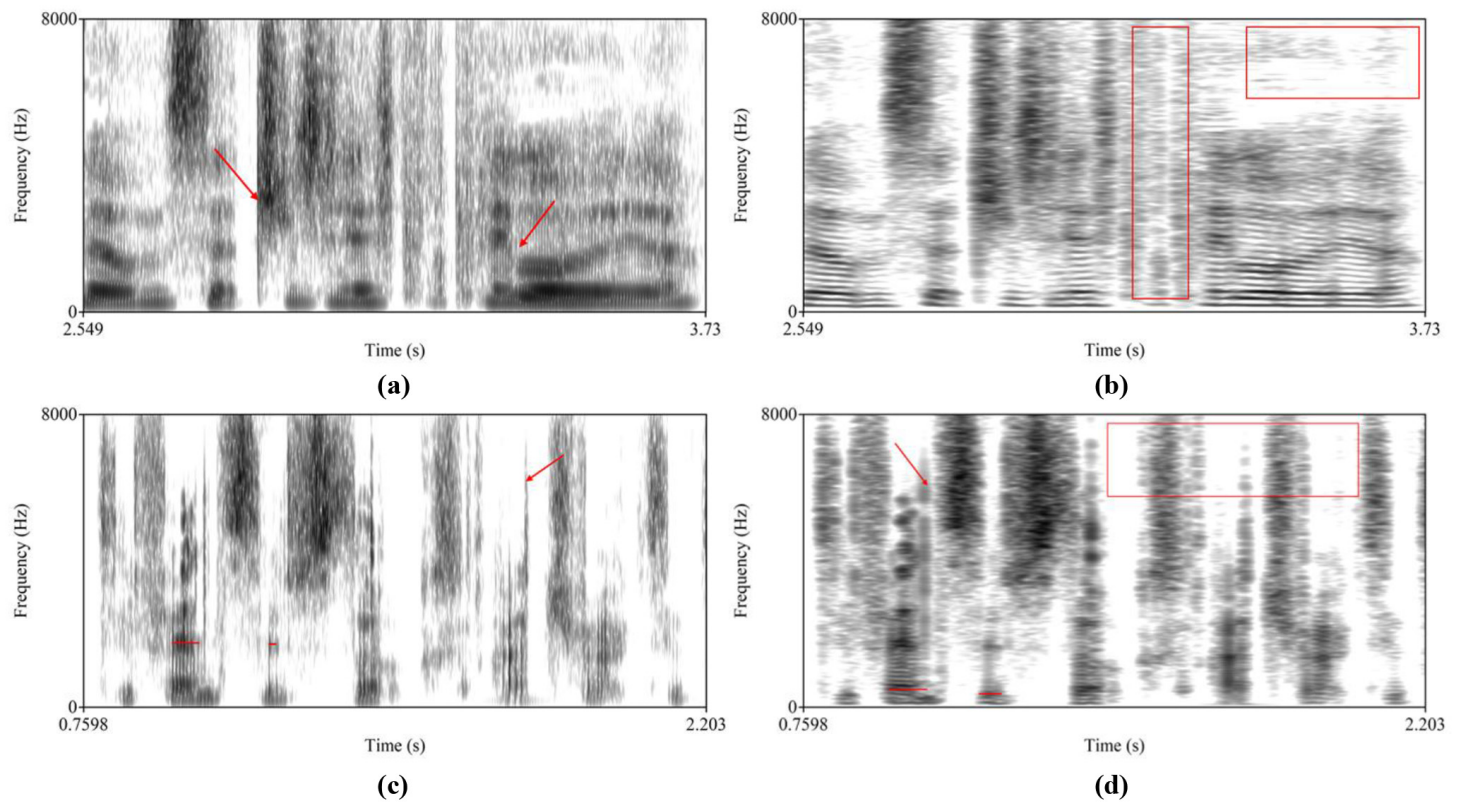
Spectrograms of deepfake speech, **(a)** wideband of a female deepfake speech, **(b)** narrowband of a female deepfake speech, **(c)** wideband of a male deepfake speech, **(d)** narrowband of a male deepfake speech.

To examine manipulated regions, wideband, and narrowband spectrograms were used to analyze deepfake audio (Fan et al., 2023). Wideband spectrograms offer excellent temporal resolution for observing temporal characteristics, while narrowband spectrograms provide favorable frequency resolution of frequency characteristics. In the Figure 1 spectrograms Figures 1a, b show wideband and narrowband representations of a female deepfake; Figures 1c, d show wideband and narrowband representations of a male deepfake. Overall, deepfake spectrograms appear blurred with dispersed energy, disconnected harmonics and unnatural patterns. In Figure 1a, the left red arrow clearly indicates concatenation artifacts; the right arrow denotes non-continuous formant misalignment. Figure 1b's left red box depicts noise interference; the right box is missing high frequencies. Figure 1c exhibits short, disconnected formants and multiple perturbations as indicated by the right red arrow. Figure 1d's red lines signify discontinuous fundamental frequencies; the red arrow denotes noise interference; the red box is missing high frequencies in several regions.

### 3.2 Linear frequency cepstral coefficient feature extraction

Certain characteristics of deepfake audio can be observed in both the temporal and frequency domains, as analyzed above. To bet-ter represent the distinguishing features of manipulated audio, acoustic features extracted for speech authentic detection should encode information from both domains. Among various acoustic representations, the LFCC features (Salim et al., 2024) of audio signals consider both temporal and spectral properties. LFCCs reflect the pattern of frequency change over time, effectively capturing characteristics of deepfakes in both the temporal and frequency domains. Compared to real speech, these features are thus capable of effectively distinguishing synthetic from natural signals for the purpose of deepfake audio detection. Encoding both temporal and spectral properties, LFCCs provide a suitable front-end representation conveying the manifestations of manipulation for downstream classification. Their joint temporal-spectral modeling facilitates capturing anomalies introduced during the deepfake generation process, offering advantageous representation for the binary classification task.

**Figure 2:**
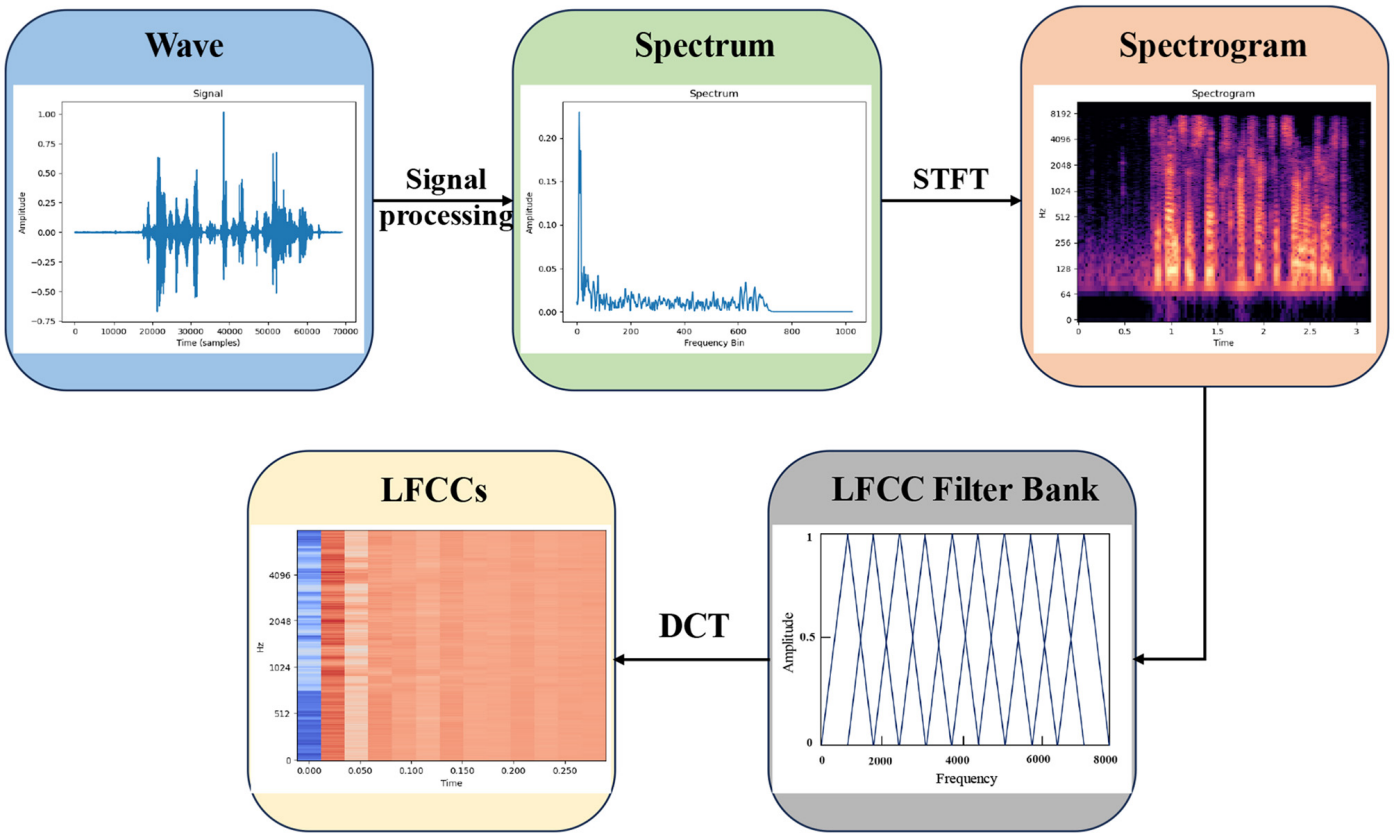
The extraction process of LFCC.

LFCC have achieved standout performance in Voice Authenticity Verification, serving as the baseline system features in the ASVspoof2019 Challenge. The extraction process of LFCC is shown in Figure 2, the audio signal is first converted from the time domain to the frequency domain by signal processing to obtain the spectrum, and the spectrogram implying the time-frequency feature is obtained by Fast Fourier Transform, and then LFCC is obtained by LFCC filter bank. The computation of these linear coefficients utilizes a linear filterbank that offers improved frequency resolution at higher bands. The LFCC filter bank expression is as follows:


(1) $$\begin{array}{lll} F_{k} & = & \overset{N-1}{\underset{n=0}{\sum }}\hat{X}\left(n\right)h_{n} \end{array}$$

(2) $$\begin{array}{lll} \hat{x}\left(n\right) & = & \left[\hat{x}\left(1\right),\hat{x}\left(2\right),\ldots ,\hat{x}\left(p\right)\right],8\leq p<16 \end{array}$$

Where *p* is the order. This LFCC representation captures both temporal and spectral characteristics of audio, facilitating the extraction of meaningful features revealing anomalies introduced during spoofing for robust fake audio detection. As evidenced by its role as the baseline system for the ASVspoof 2019 Challenge, LFCC has proven highly effective at this critical task. In this study, LFCCs demonstrably outperform both MFCCs and GFCCs (Wu et al., 2014). By employing uniformly spaced linear filter banks instead of Mel's nonlinear scale, LFCCs achieve superior spectral resolution at high frequencies, sensitively capturing the artifacts characteristic of deepfake synthesis. Moreover, the cepstral transform at the heart of LFCCs not only preserves the full short-term energy distribution but, through framewise sliding windows, precisely tracks dynamic spectral changes over time thereby revealing temporal discontinuities that GFCCs, which focus primarily on spectral-envelope energy, fail to detect. Crucially, LFCCs inherently fuse spectral-envelope and cepstral-phase information, enabling downstream classifiers to harness complementary temporal and spectral cues for enhanced detection performance (Lavrentyeva et al., 2017).

### 3.3 Proposed model

In Voice Authenticity Verification tasks, CNN networks demonstrate strong spectral learning ability through extracting manipulation cues from synthesized speech, but lack the ability to mine temporal speech information. Long Short Term Memory (LSTM) networks can capture speech temporal patterns but have weak spectral learning for acoustic signals. A hybrid CNN-LSTM architecture that leverages their complementary strengths can alleviate individual limitations and boost detection performance. A single network often struggles to capture global characteristics of target objects in complex detection systems. Model integrates multiple neural network types into a unified model to enhance both prediction accuracy and generalization of the detector. To achieve optimal detection performance, each constituent neural network in an ensemble ideally possesses some level of capability while also exhibiting diversity and complementary strengths. Networks with differing architectures, optimizations, or input domains are commonly combined to leverage their respective advantages. The detail of proposed model is shown in Table 1.

**Table 1:** The detail of proposed model.

**Layers**	**Output**	**Paramenters**
Conv2D	(None, 48, 18, 32)	320
Conv2D	(None, 46, 16, 64)	18,496
MaxPooling	(None, 23, 8, 64)	0
Dropout	(None, 23, 8, 64)	0
Conv2D	(None, 21, 6, 64)	36,928
MaxPooling	(None, 9, 2, 128)	0
Batch Normalization	(None,9,2,128)	512
Flatten	(None, 2,304)	0
Reshape	(None, 9, 256)	0
Dense	(None, 9, 64)	16,448
LSTM	(None, 9, 64)	33,024
LSTM	(None, 128)	98,816
Dropout	(None, 128)	0
Batch Normalization	(None, 128)	512
Dense	(None, 256)	33,024
Dropout	(None, 256)	0
Batch normalization	(None, 256)	1,024
Dense	(None, 2)	514

**Figure 3:**
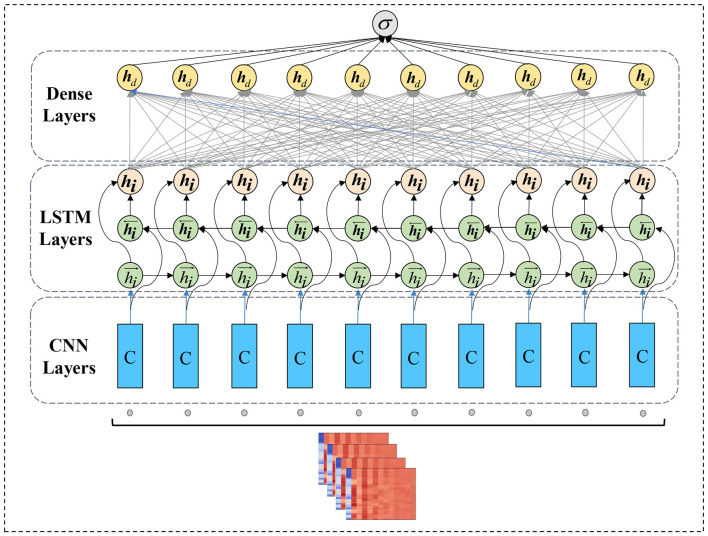
The proposed model structure.

For example, a convolutional network may extract spatial features, while recurrent network models temporal dynamics. By fusing varied but correlated decision perspectives, ensemble methods can distill more comprehensive representations than any independent component, mitigating their individual blind spots and noise to realize emergent synergistic effects greater than the sum of parts. This multipronged modeling approach has proven effective for various audio and visual analysis challenges by aggregating diverse learned abstractions into a robust integrated system. The proposed model structure is shown in Figure 3.

The proposed model contains 18 layers. The input feature sequence dimension is (*X*, 50, 20, 1), where *X* represents the number of feature maps. The first layer is a convolutional layer with 32 neurons, accepting inputs of size 50 × 20 × 1 via 3 × 3 filters and using ReLU activation. The second convolutional layer has 64 neurons and 3 × 3 filters with ReLU activation. After two convolutional operations, the third layer applies 2 × 2 max pooling, followed by 50% dropout for regularization. The fifth layer adds another convolutional layer with 64 neurons and 3 × 3 filters, activated by ReLU. The sixth layer performs max pooling. After batch normalization in the seventh layer, the data is flattened and reshaped before dense layers. The reshaped data then enters the LSTM part of the hybrid network, containing two recurrent layers with 64 and 128 hidden units respectively, each using ReLU activation. Dropout and batch normalization are applied.The 15th layer contains two dense blocks, with the first having 256 neurons and ReLU activation, while the second performs softmax classification for the target variable. Dropout and batch normalization layers are inserted between the dense blocks. This CNN-LSTM architecture effectively leverages both spectral and temporal modeling capabilities.

#### 3.3.1 CNN

**Figure 4:**
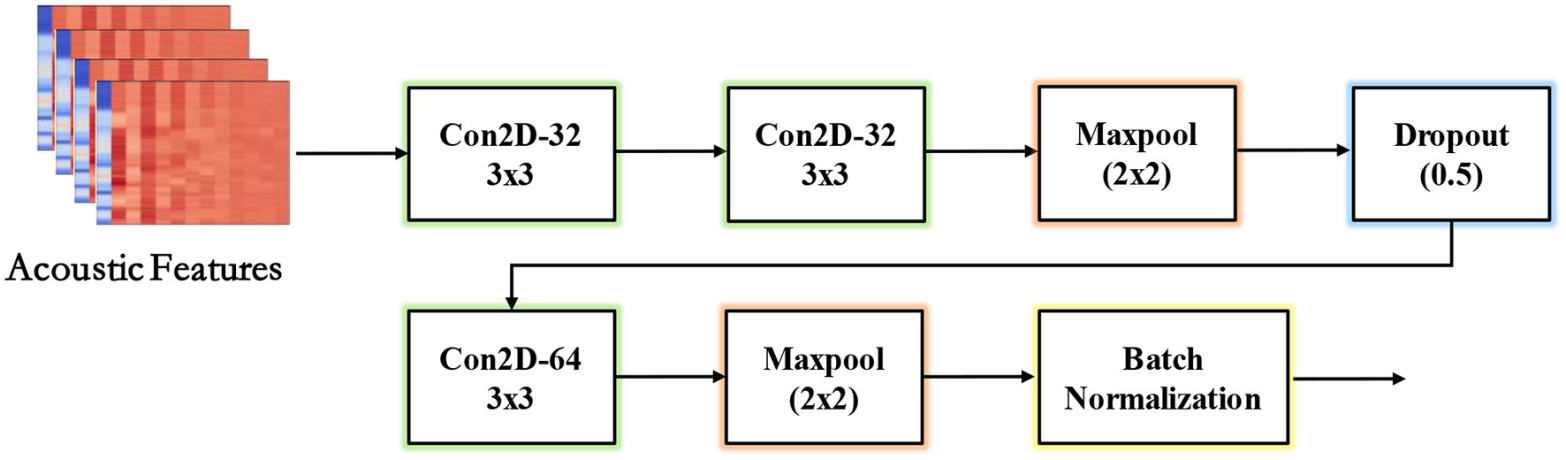
The basic architecture of our CNN structure.

Convolutional neural networks (CNNs) are a class of feedforward neural networks widely used in domains such as face detection, speech recognition, and activity recognition (Jin et al., 2022, 2024). Standard CNNs consist of convolutional layers, pooling layers, fully connected layers and activation functions. Convolutional layers contain trainable filters to extract features from input data. Pooling layers enhance translation invariance by reducing the spatial resolution of feature maps. Fully connected layers act as classifiers, while common activation functions include Sigmoid, Tanh, and ReLU. Figure 4 depicts the basic architecture of our CNN structure, which computations can be expressed as equation:


(3) $$\begin{array}{c} y_{i}=f\left(b_{i}+\overset{N}{\underset{i=0}{\sum }}k_{ij}*x_{i}\right) \end{array}$$

Where * denotes the convolutional operation, *f* represents activate function, *b* denotes the bias term and *x*_*i*_ is the input vector.

This convolutional filtering enables the model to automatically learn high-level audio representations directly from input acoustic sequences. Through successive convolutional layers interspersed with activation and pooling operations, insights into temporal patterns indicative of manipulation vs. natural speech signals can be distilled for final classification or regression. The end-to-end CNN framework is wellsuited for this domain thanks to its support of sequential data.

#### 3.3.2 LSTM

LSTM networks were developed by Hochreiter and Schmidhuber in 1997 to address the issue of vanishing gradients in traditional RNNs (Sundermeyer et al., 2012). LSTMs introduce a gating mechanism that controls the flow of information and a memory cell that stores state, preventing early signals from decaying during processing. While LSTMs alleviate the gradient problem of RNNs to some extent, individual neural units handling four linear layers means increased parameters and computation as network depth grows, easily leading to overfitting.

LSTMs comprise a series of memory cells that typically contain a self-connected memory cell to store the network's temporal state information. LSTMs have three gates input, output and forget—that regulate the flow of information. The input gate deter-mines what new information is stored in the cell; the output gate determines what cell state values are output; and the forget gate determines what should be forgotten from the cell state.

**Figure 5:**
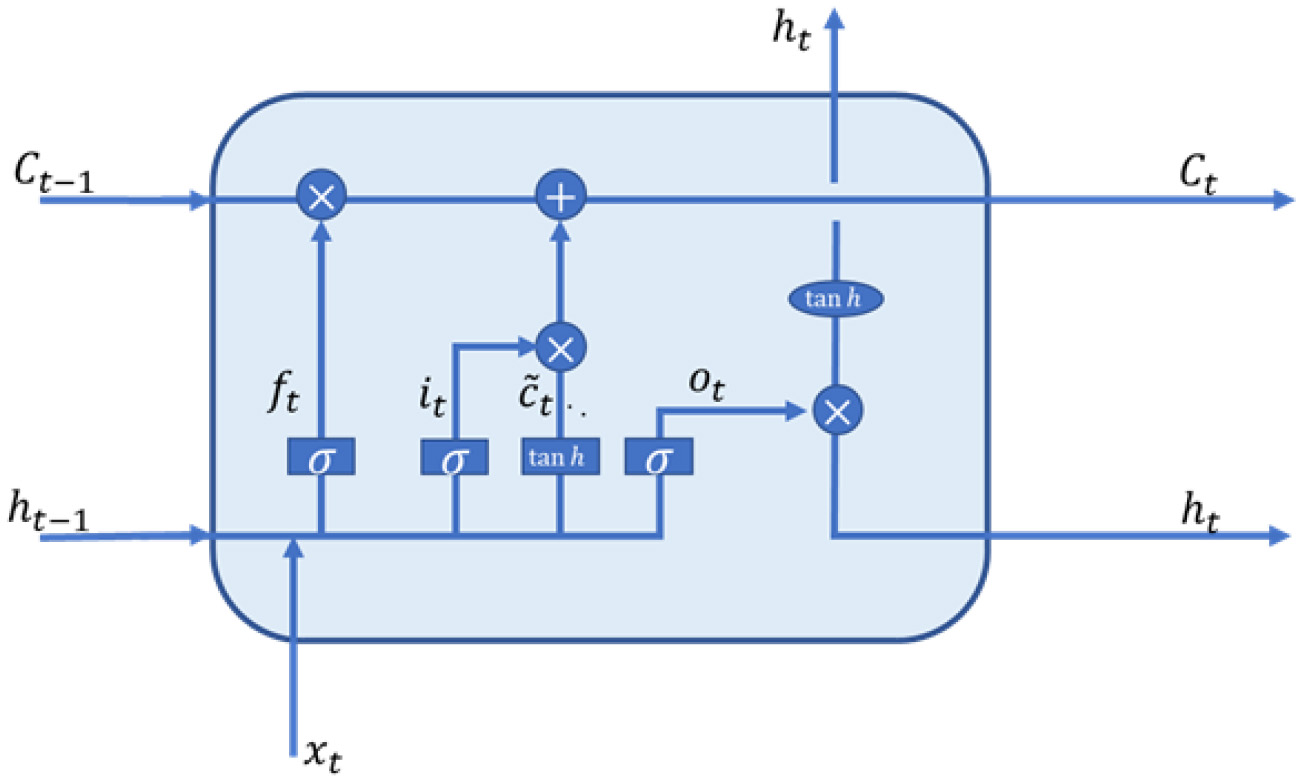
LSTM memory cell structure.

As shown in Figure 5, an LSTM memory cell at time step t can be represented by the following equations:


(4) $$\begin{array}{c} f_{t}=sW_{f}\left[h_{t-1},x_{t}\right]+b_{f} \end{array}$$

(5) $$\begin{array}{c} i_{t}=s\left(W_{i}\left[h_{t-1},x_{t}\right]+b_{c}\right) \end{array}$$

(6) $$\begin{array}{c} {\tilde{C}}_{t}=\tanh\left(W_{c}\left[h_{t-1},x_{t}\right]+b_{c}\right) \end{array}$$

(7) $$\begin{array}{c} C_{t}=f_{t}*C_{t-1}+i_{t}*{\tilde{C}}_{t} \end{array}$$

(8) $$\begin{array}{c} o_{t}=s\left(W_{o}\left[h_{t-1},x_{t}\right]+b_{o}\right) \end{array}$$

(9) $$\begin{array}{c} h_{t}=o_{t}*\tanh\left(C_{t}\right) \end{array}$$

In this equation, where *s* is the state vector, where *C*_*t*−*l*_, *h*_*t*−*l*_ are the internal state of the previous LSTM memory cell and the external state of the hidden layer, respectively. *x*_*i*_ is the input speech signal feature sequence, σ is the activation function sigmoid, $i_{t},o_{t},f_{t},C_{t},{\tilde{C}}_{t}$ are denote as input gate, output gate, forgetting gate, content of memory cell and content of new memory cell, respectively. *W*_*f*_, *W*_*i*_, *W*_*c*_, *W*_*o*_ denote the weight matrices of the forgetting gate, the input gate, the content of memory cell, and the output gate, respectively and *b*_*f*_, *b*_*c*_, *b*_*o*_ denote the bias vectors of the forgetting gate, the content of memory cell, and the output gate, respectively.

## 4 Experiments

### 4.1 Dataset and pre-processing

The experiments were conducted utilizing the ASVspoof 2019 database (Nautsch et al., 2021), which is an English speech corpus constructed upon the VCTK library comprising natural and synthetic speech samples, organized into LA and PA subsets. The study investigated speech synthesis and voice conversion attacks using the LA subset. This dataset is partitioned into training, development, and evaluation sets for structural coherence, with the training set containing 25,380 speech samples, the development set 24,844, and evaluation set 71,237, as detailed in Table 2, and there is no speaker overlap between subsets.

**Table 2:** Detail of the ASVspoof2019LA dataset.

**Dataset**	**Sex of speaker**		**Phonetic character**	
	**Male**	**Female**	**Authentic**	**Synthetic**
Train	8	12	25,800	22,800
Development	4	6	22,480	22,296
Evaluation	21	27	63,550	63,882

The experimental environment involved an Ubuntu 18.04.4 LTS system within the Jupyter Notebook development environment, configuring Python version 3.6 coupled with the TensorFlow 2.0 deep learning framework. Hardware-wise, an Intel Xeon(R) Gold 6,132 processor was leveraged alongside multiple NVIDIA Tesla P4 graphics processing units and 125.3 GiB of memory to facilitate deep learning experimentation and kernel deployment over the GPU cluster.

For any individual speech signal within the database, a frame-based method was adopted to extract multiple acoustic feature sequences, with each sequence composed of 50 frames and 20-dimensional features. Redundant sequences were discarded, and labels were sequentially assigned to the remaining multisegment feature sequences. This preprocessing scheme aimed to strike a balance between granularity and computational feasibility for the ensuing recurrent classification task, segmenting the variable-length in-puts into fixed-size temporal windows matching the network architecture while retaining necessary identity information distributed across the sequential and multi-channel inputs.

To further validate the model's generalization ability, we conducted additional experiments on the WaveFake dataset (Frank and Schönherr, 2021). This dataset consists of approximately 93,000 utterances of 16 kHz single-channel speech, including genuine samples from LibriSpeech and forged samples generated by six mainstream neural vocoders: WaveNet, WaveGlow, MelGAN, Parallel WaveGAN, MB-MelGAN, and LPCNet. However, the experimental protocol does not clearly distinguish between in-domain and cross-domain test-ing, which may lead to an inadequate interpretation of the model's generalization performance.

To address this, we trained the model on the ASVspoof2019 LA dataset and validated it on the WaveFake dataset without any fine-tuning, aiming to test the model's robustness against unseen forgery attacks. This cross-dataset training and testing approach simulates the model's performance when encountering novel vocoders and synthesis methods in real-world scenarios. Future work will focus on clearly defining in-domain vs. cross-domain testing to provide a more accurate evaluation of model generalization.

### 4.2 Evaluation metrics and implementation details

In this paper we use F1 score, EER, and AUC as evaluation metrics. For binary classification algorithms, the detection results of the algorithm are categorized into four classes based on the combination of predicted values and actual values: true positives (TP), false positives (FP), true negatives (TN), and false negatives (FN). All possible outcomes are presented in the form of a confusion matrix, as shown in Table 3.

**Table 3:** Confusion matrix of possible outcomes.

	**Authentic**	**Synthetic**
Authentic	TP	FN
Synthetic	FP	TN

F1 score is a commonly used metric in binary classification algorithms to comprehensively evaluate the robustness of a model. The essence of the F1 score is the harmonic mean of precision and recall, ranging from 0 to 1. A value closer to 1 indicates better performance of the model, while a value closer to 0 indicates poorer performance. The mathematical expression of the F1 score is shown below:


(10) $$\begin{array}{c} F_{1}=\frac{2\cdot \text{Precision}\cdot \text{Recall}}{\text{Precision}+\text{Recall}} \end{array}$$

Precision refers to the proportion of correctly predicted natural speech samples among all samples predicted as natural speech, while Recall refers to the proportion of correctly predicted natural speech samples among all samples that are actually natural speech. The mathematical formulas for both are as follows.


(11) $$\begin{array}{cc} Precision & =\frac{TP}{TP+FP} \end{array}$$

(12) $$\begin{array}{cc} Recall & =\frac{TP}{TP+FN} \end{array}$$

Speech authentic detection algorithm usually uses equal error rate as an evaluation metric. Equal error rate is a metric used to predetermine the thresholds of False Acceptance Rate (FAR) and False Rejection Rate (FRR). In speech attack detection, FAR refers to the probability of synthetic speech being incorrectly accepted as natural speech and FRR refers to the probability of natural speech being incorrectly rejected as synthetic speech. When FAR and FRR are equal, this equal value is called equal error rate, which is mathematically defined as follows:


(13) $$\begin{array}{c} FAR\left(\theta \right)=\frac{FP}{TP+FP} \end{array}$$

(14) $$\begin{array}{c} FRR\left(\theta \right)=\frac{FN}{TN+FN} \end{array}$$

(15) $$\begin{array}{c} EER=FAR\left(\theta_{EER}\right)=FRR\left(\theta_{EER}\right) \end{array}$$

θ is used as the determination threshold of the detection system, when the prediction value of the input speech is greater than θ, the test speech is determined as authentic speech, and conversely when the prediction value of the input speech is less than θ, the test speech is determined as synthetic speech. The higher the threshold θ, the stricter the acceptance conditions of the system, the lower the value of the corresponding FAR, and the higher the value of FRR. the smaller the value of EER, the stronger the detection algorithm distinguishes between natural and synthetic speech, and the better the performance of the detection system. The Detection Error Trade-offs Curve (DET) is usually used to reflect the relationship between FAR and FRR, with FAR as the horizontal coordinate value and FRR as the vertical coordinate value, and the threshold is adjusted to determine the relationship between the two to form the DET curve, while the intersection of the DET curve with the diagonal of the first quadrant is the value of EER.

Area under the Curve of ROC (AUC) is an evaluation metric often used in binary classification tasks, which refers to the area under the ROC curve (Koenig and Lacey, 2015). The ROC curve can be used to evaluate the performance of a classification model by means of two metrics, namely the true case rate and the false positive rate, and the closer the AUC is to 1 means the better the model is. The formula for calculating the AUC is as follows:


(16) $$\begin{array}{c} AUC=\frac{\sum_{i\in \text{positiveClass}}{\mathrm{rank}}_{i}-\frac{M\left(1+M\right)}{2}}{M\cdot N} \end{array}$$

Where rank_*i*_ represents the serial number of the ith sample, M is the number of positive samples and N is the number of negative samples.

Table 4 summarizes the main training hyperparameters for our proposed methods.

**Table 4:** Hyperparameters.

**Config**	**Value**
Optimizer	Adam
Learning rate	1e − 4
epoch	100
Batch size	16
Scheduler	CosineAnnealingLR

## 5 Results

### 5.1 Model comparison

**Figure 6:**
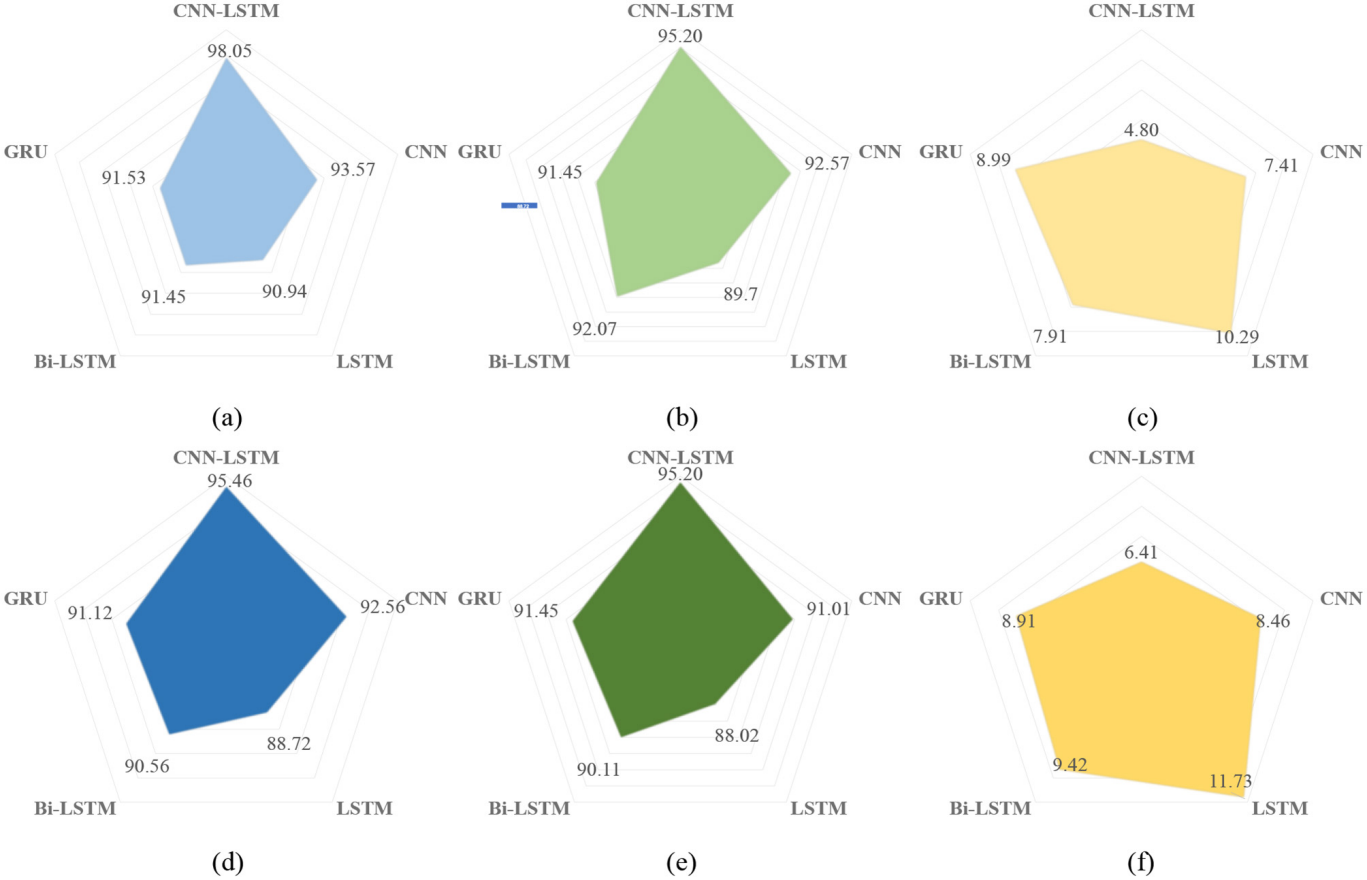
Radar chart of results. **(a)** Results of five models on Accuracy using ASVspoof2019LA, **(b)** results of five models on AUC using ASVspoof2019LA, **(c)** results of five models on EER using ASVspoof2019LA, **(d)** results of five models on Accuracy using Wavefake, **(e)** results of five models on AUC using Wavefake, **(f)** results of five models on EER using Wavefake.

To better evaluate the effectiveness of the proposed CNN-LSTM method for forensic speech authentic detection, a controlled experiment was designed with a comparison group. Four neural network models were selected for comparative analysis. Each network model was configured with identical parameters, and the input acoustic features were uniformly LFCC. The experimental process rigorously controlled for singular variables, and the results are presented in the Table 5, and we also use radar chart to visualize the results and is shown in Figure 6.

**Table 5:** The result of comparative analysis.

**Model**	**ASVspoof2019LA**			**Wavefake**		
	**Accuracy(%)**	**AUC(%)**	**EER(%)**	**Accuracy(%)**	**AUC(%)**	**EER(%)**
CNN	93.57	92.57	7.41	92.56	91.01	8.46
LSTM	90.94	89.70	10.29	88.72	88.02	11.73
Bi-LSTM	91.45	92.07	7.91	90.56	90.11	9.42
GRU	91.53	91.00	8.99	91.12	90.75	8.91
ASSERT (Lai et al., 2019)	94.00	93.13	6.70	93.20	92.51	7.98
RawNet2 (Tak et al., 2021)	91.24	90.56	9.50	89.87	89.51	9.67
CNN-LSTM	**98.05**	**95.20**	**4.80**	**95.46**	**93.82**	**6.41**

Bold indicates the best value.

The CNN–LSTM architecture demonstrates clear superiority over all baseline models across both datasets. On ASVspoof2019-LA, it achieves an impressive accuracy of 98.05%, AUC of 95.20%, and a low EER of 4.80%. Its performance remains strong on the more challenging WaveFake dataset, with 95.46% accuracy, 93.82% AUC, and 6.41% EER. In comparison, the standalone CNN model, although maintaining decent accuracy (93.57% on ASVspoof2019-LA and 92.56% on WaveFake), exhibits higher error rates (7.41% and 8.46%, respectively), indicating its limitations in capturing the temporal dependencies required for deepfake detection. Similarly, LSTM, Bi-LSTM, and GRU models show lower performance, with accuracy around 90%–91.5% and higher EERs ranging from 8.99% to 11.73%, reflecting the inadequacy of sequence-based models in capturing the complex spectro-temporal patterns of deepfake audio. ASSERT shows competitive results, with 94.00% accuracy on ASVspoof2019-LA and 93.20% on WaveFake, but its EER values are relatively higher (6.70% and 7.98%). RawNet2, while demonstrating 91.24% accuracy on ASVspoof2019-LA and 89.87% on WaveFake, has EERs of 9.50% and 9.67%, respectively, indicating its slightly less efficient performance compared to CNN–LSTM.

Although all models experience a small decline in performance when transitioning from ASVspoof2019-LA to WaveFake (for in-stance, CNN–LSTM's accuracy decreases by 2.6%, and its EER rises by 1.6%), the relative rankings remain stable, underlining the robustness and adaptability of the CNN–LSTM model across datasets. These results confirm that integrating convolutional feature extraction with recurrent temporal modeling is crucial for achieving both high detection accuracy and resilience across different deepfake audio scenarios.

The cross-domain validation experiment results shown in Table 6, all models exhibited varying degrees of performance degradation compared to their results on single-dataset evaluations, highlighting the challenges posed by unseen spoofing types to model generalization. Specifically, the CNN–LSTM model achieved the highest accuracy (69.87%) and the lowest EER (38.85%), significantly outperforming the other models. This indicates that the hybrid architecture, which combines convolutional feature extraction with recurrent temporal modeling, effectively captures cross-dataset commonalities in deepfake audio and offers strong transferability. In contrast, standalone CNN and recurrent neural networks (LSTM, Bi-LSTM, GRU) showed similar performance, with accuracies ranging from 59.89% to 62.55% and relatively high EERs (42.14% to 45.00%), suggesting that single-mode feature extraction is insufficient for complex cross-domain detection tasks. Moreover, while the Bi-LSTM demonstrated slight improvements over the unidirectional LSTM in both accuracy and EER, the gains were limited. These findings further validate the importance of composite feature modeling strategies in enhancing the robustness of speech authentic detection systems. Overall, the results demonstrate that the CNN–LSTM architecture provides superior generalization when confronted with previously unseen spoofing types, making it a strong candidate for complex audio forgery detection tasks.

**Table 6:** Out of domain experiment.

**Model**	**Accuracy**	**EER (%)**
CNN	62.55	42.14
LSTM	59.89	45.00
Bi-LSTM	61.46	43.31
GRU	60.87	44.52
CNN-LSTM	**69.87**	**38.85**

The training set is ASVspoof2019, the testing set is Wavefake; Bold indicates the best value.

**Figure 7:**
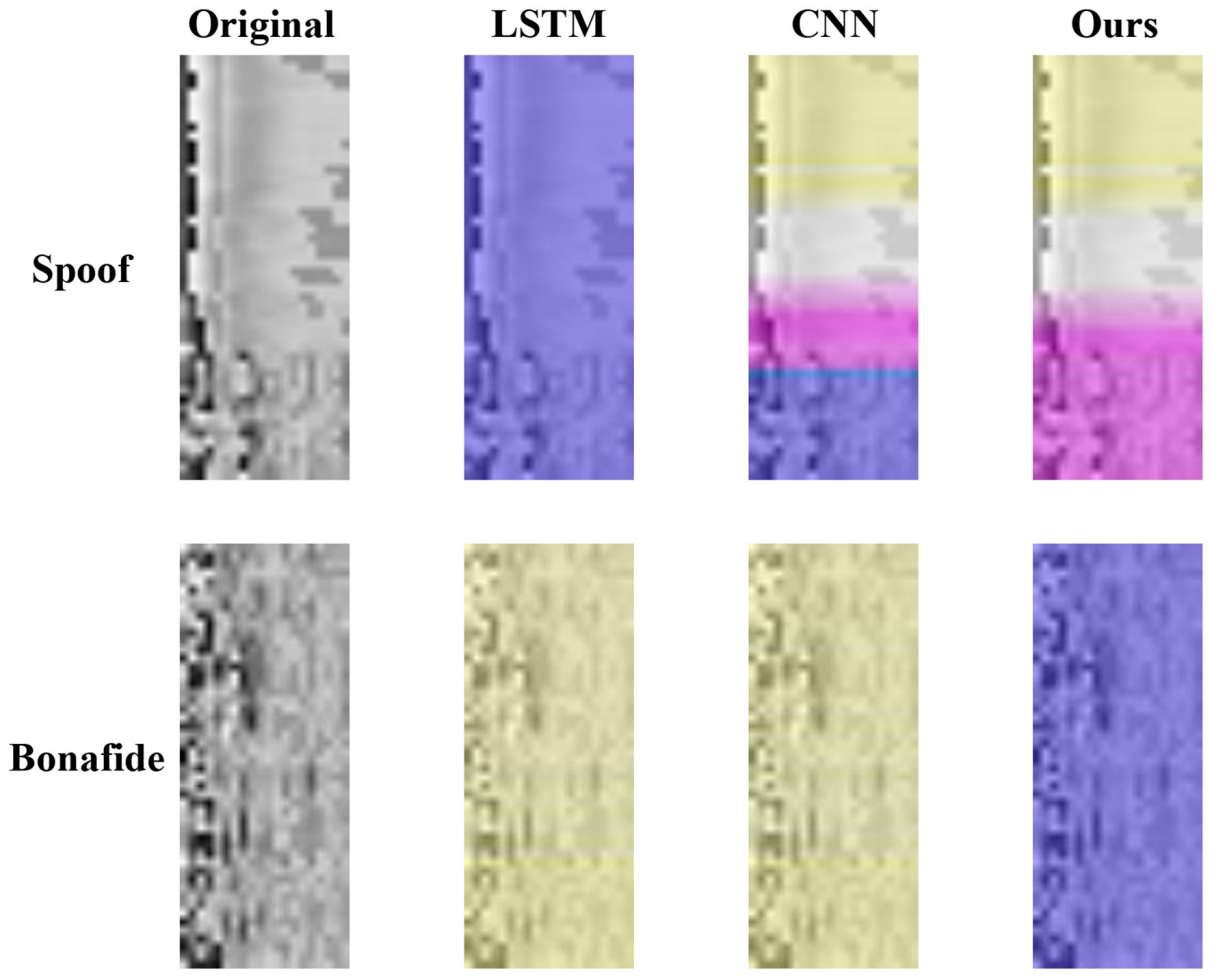
Grad-CAM map for the CNN, LSTM and CNN–LSTM models.

To further examine the decision-making basis of the models and the impact of different architectures, we applied Grad-CAM to visualize the regions of input features that contributed the most to the classification outcomes shown in Figure 7. The top and bottom rows correspond to genuine and deepfake speech samples, respectively, while each column presents the original input features alongside Grad-CAM heatmaps for the CNN, LSTM, and CNN–LSTM models.

The CNN model primarily targets low-frequency regions where energy concentrations are strongest, effectively capturing basic spectral patterns but showing limited sensitivity to temporal dynamics. In contrast, the LSTM model focuses on temporal anomalies within the sequence but lacks responsiveness to spatial features such as high-frequency artifacts. Notably, the CNN–LSTM model, by integrating convolutional spatial feature extraction with recurrent temporal modeling, exhibits a balanced attention pattern: it highlights stable mid-to-low frequency regions in genuine speech, while accurately identifying high-frequency anomalies and spectral discontinuities in deepfake samples.

The CNN-LSTM attention maps align closely with the high-frequency and temporal variation regions encoded by the LFCC features, confirming the model's superior capacity to jointly capture both spectral and temporal characteristics. These findings indicate that the CNN–LSTM model not only detects static spectral irregularities but also effectively captures disruptions to temporal continuity introduced during synthesis—demonstrating clear advantages in both detection accuracy and generalization over the standalone CNN and LSTM architectures.

### 5.2 Acoustic

To assess the effectiveness of various acoustic features in the proposed model, we selected five prominent features: Mel-frequency cepstral coefficients (MFCC), Gammatone-frequency cepstral coefficients (GFCC), Filterbank features (Fbank), Spectrogram, and Liftered Mel-frequency cepstral coefficients (LFCC). While MFCC and GFCC share similarities with LFCC in their signal pro-cessing approaches, they differ primarily in filterbank design, leading to distinct applications.

MFCC is widely used in speaker recognition tasks due to its strong ability to capture speaker-specific characteristics. On the other hand, GFCC is known for its robustness and suitability for acoustic tasks in complex environments, owing to its ability to handle a wide range of acoustic conditions. Fbank and Spectrogram are often used in speech recognition due to their ability to represent temporal and spectral information. Specifically, Fbank is effective at capturing spectral features in a more compact form, while Spectrogram provides a detailed time-frequency representation. LFCC, with its focus on the high-frequency band of audio signals, is recognized for its anti-spoofing capabilities, making it particularly useful in deepfake speech detection, where verifying authenticity is essential.

In the experimental setup, we varied the acoustic features while keeping all other variables constant to ensure a fair comparison. The results, as summarized in Table 7, are evaluated using key performance metrics such as EER%, Accuracy%, AUC%, and F1-Score for both authentic and synthetic speech data.

**Table 7:** The result of acoustics comparative experiments.

**Feature**	**EER%**	**Accuracy%**	**AUC%**	**F1-Score**	
				**Authentic**	**Synthetic**
MFCC	5.79	97.42	93.21	0.88	**0.99**
GFCC	7.54	97.24	92.45	0.86	0.98
Fbank	6.33	97.32	92.64	0.87	0.98
Spectrogram	8.62	96.23	91.15	0.85	0.96
LFCC	**4.80**	**98.05**	**95.20**	**0.89**	**0.99**

Bold indicates the best value.

From the table above, LFCC outperforms other features, achieving the lowest EER% of 4.80% and the highest accuracy of 98.05%, AUC% of 95.20%, and F1-Score of 0.99, making it highly effective for deepfake speech detection. MFCC follows closely with strong performance, particularly in accuracy of 97.42% and AUC% of 93.21%. GFCC shows solid robustness, with an EER% of 7.54% and accuracy of 97.24%. Fbank and Spectrogram perform comparatively lower, with Spectrogram showing the highest EER% of 8.62% and the lowest accuracy of 96.23%.

**Figure 8:**
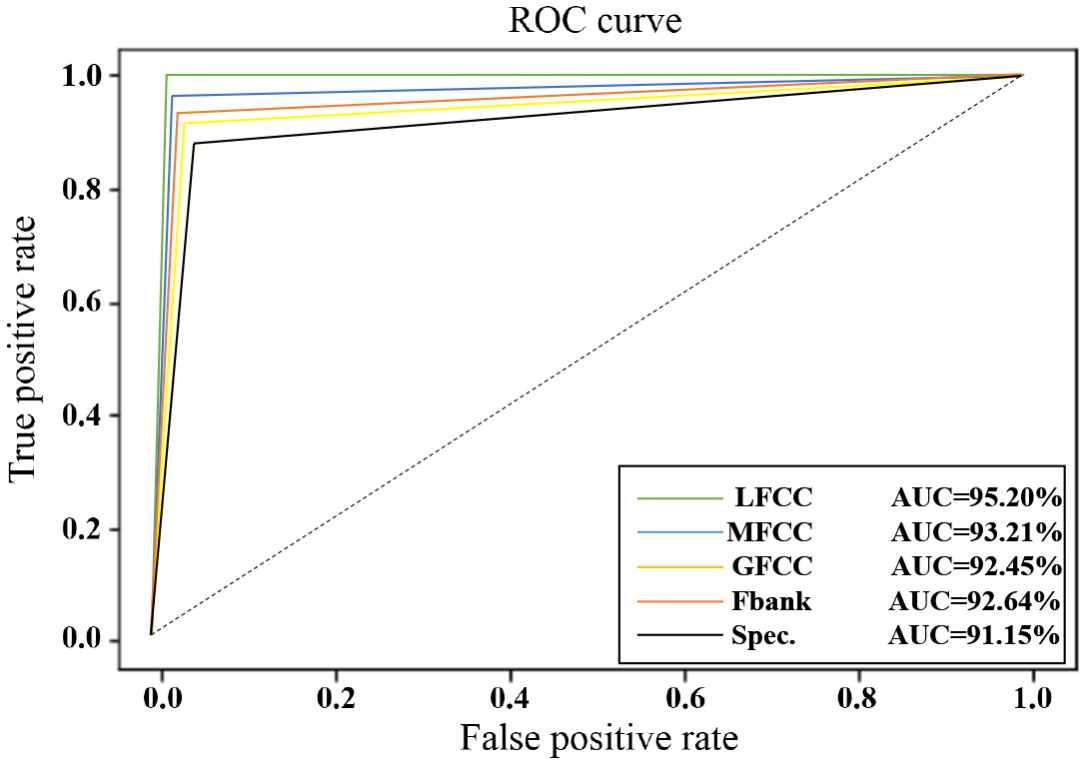
The ROC curves of the three types of features.

Figure 8 depicts the ROC curves for five types of features on the CNN-LSTM model, providing a visual representation of their respective AUC values. It is observed that the area under the ROC curve is largest for LFCC features, followed by MFCC, Fbank, GFCC, and Spectrogram, indicating their descending order of performance in terms of AUC. This visual representation reaffirms that LFCC features exhibit the highest discrimination ability between authentic and synthetic speech, making them the most effective for tasks requiring precise authenticity verification in audio signals.

**Figure 9:**
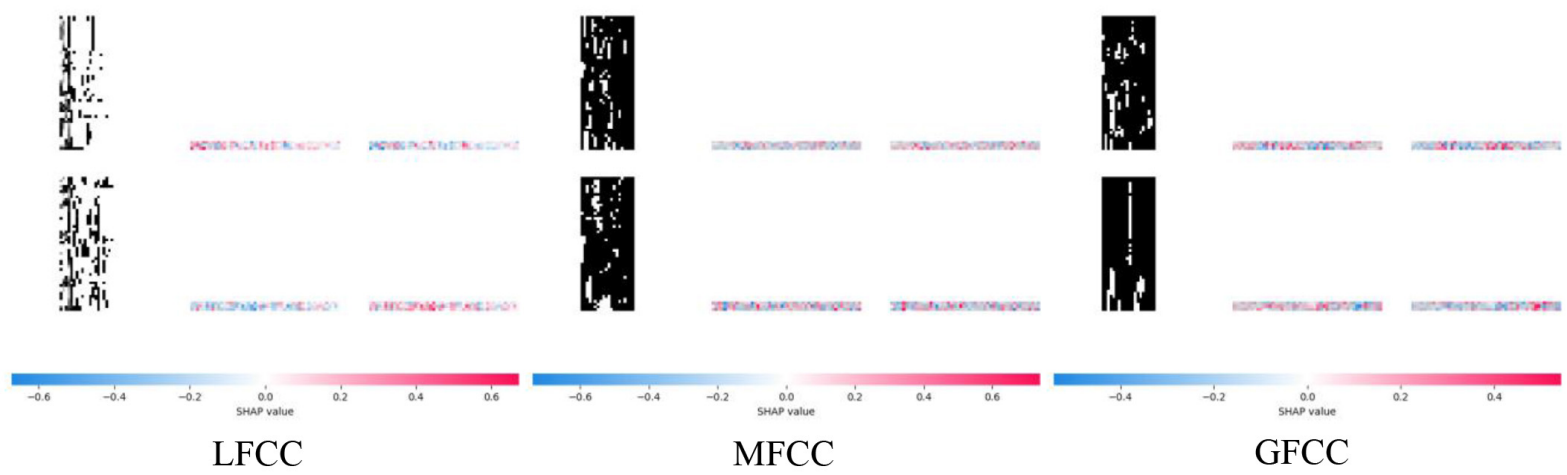
The SHAP plot of the three types of features.

To further elucidate the contributions of different acoustic features to model decisions, we conducted a SHAP (SHapley Additive Explanations) analysis for MFCC, LFCC, and GFCC features shown in Figure 9.

The results reveal that LFCC features exhibit a distinct and interpretable SHAP pattern, with significant positive contributions (indicated by red regions) concentrated in the high-frequency and spectral variation areas of spoofed samples. In contrast, SHAP values for genuine samples are more uniformly distributed and predominantly negative, suggesting that LFCC effectively highlights the anomalies inherent in manipulated audio and serves as the primary basis for the model's predictions. By comparison, MFCC and GFCC features display more scattered SHAP distributions with mixed positive and negative contributions, particularly for fake samples, where SHAP values largely hover around zero. This indicates their limited utility in revealing artifacts associated with deep-fake generation.

Furthermore, the clear divergence in SHAP distributions between genuine and fake samples underscores LFCC's role in providing stable and interpretable decision cues. These findings further validate LFCC as the preferred acoustic representation for speech authentic detection and explain why the CNN–LSTM model consistently outperforms standalone CNN or LSTM architectures—its decision process effectively leverages the time-frequency anomaly information encoded within LFCC features, enhancing both accuracy and generalization.

**Figure 10:**
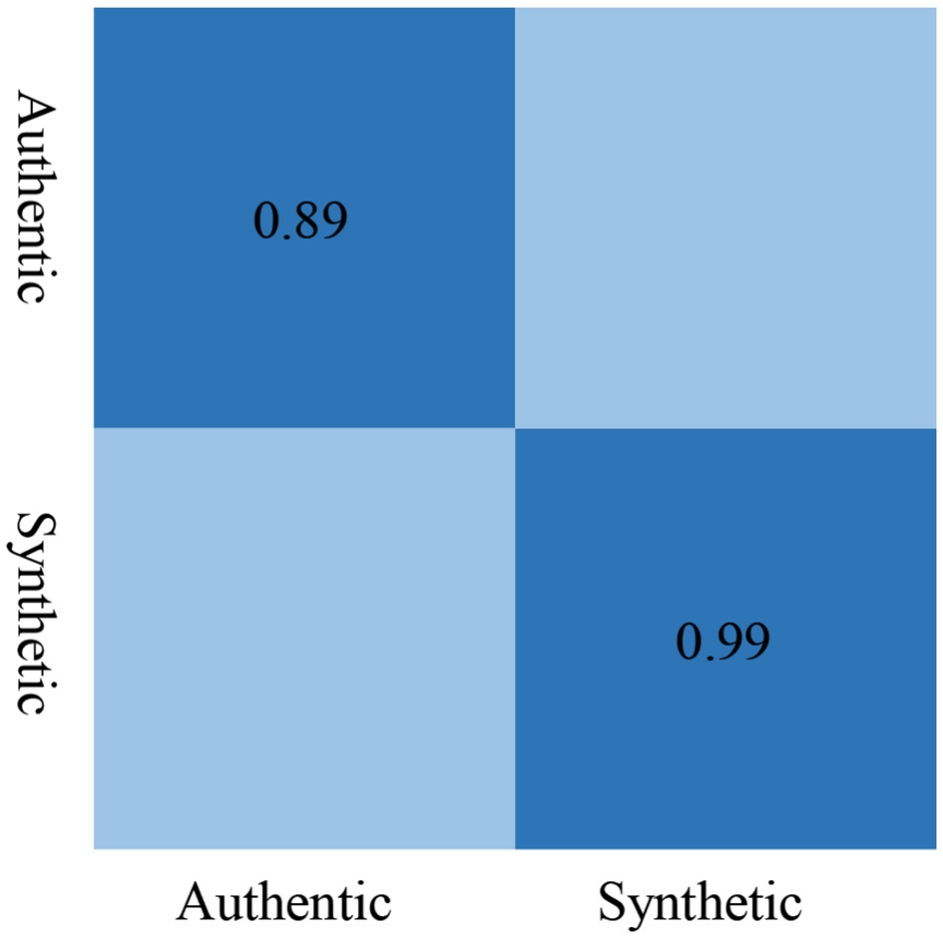
Confusion matrix of F1-score.

We used a confusion matrix Figure 10 to provide a more intuitive view of the F1-Score metrics. It is observed that when using LFCC features, the F1 Score for detecting authentic speech is 0.89, and for detecting synthetic speech is 0.99, which is the highest among the three types of features.

This demonstrates that LFCC features excel in both precision and recall for identifying both authentic and synthetic speech instances, making them the most effective choice among the evaluated acoustic features for this task.

## 6 Conclusions

In this study, we developed and validated an explainable CNN–LSTM fusion model for forensic speech authentication, leveraging LFCC features to jointly capture spectral and temporal properties of audio. Experimental results on ASVspoof2019 LA and WaveFake confirm that the proposed model surpasses conventional CNN, LSTM, and recurrent variants in accuracy, AUC, and EER, while demonstrating robust cross-dataset generalization. Beyond performance, explainable AI techniques such as Grad-CAM and SHAP revealed interpretable decision bases, showing that the model's focus on high-frequency artifacts and temporal inconsistencies parallels cognitive auditory mechanisms underlying human speech perception.

By embedding model interpretability within a cognitive neuroscience perspective, this work advances the development of trustworthy forensic tools that not only detect deepfakes but also provide transparent reasoning for expert evaluation in legal proceedings. Future research will explore tighter integration of psychoacoustic sensitivity profiles, multimodal cues, and neuro-inspired front ends to further enhance robustness and cognitive plausibility. These findings highlight the potential of explainable and cognitively grounded AI methods as a bridge between computational neuroscience and forensic audio analysis, contributing to both scientific understanding and evidentiary practice.

## Data Availability

The raw data supporting the conclusions of this article will be made available by the authors, without undue reservation.
